# Stability and Selective Vapor Sensing of Structurally Colored Lepidopteran Wings Under Humid Conditions

**DOI:** 10.3390/s20113258

**Published:** 2020-06-08

**Authors:** Gábor Piszter, Krisztián Kertész, Zsolt Bálint, László Péter Biró

**Affiliations:** 1 Institute of Technical Physics and Materials Science, Centre for Energy Research, P.O. Box 49, H-1525 Budapest, Hungary; kertesz@mfa.kfki.hu (K.K.); biro@mfa.kfki.hu (L.P.B.); 2 Hungarian Natural History Museum, 13 Baross St., H-1088 Budapest, Hungary; balint.zsolt@nhmus.hu

**Keywords:** butterfly wing, moth wing, photonic nanoarchitecture, vapor sensing, chemical selectivity, optical readout, principal component analysis, humidity

## Abstract

Biological photonic nanoarchitectures are capable of rapidly and chemically selectively sensing volatile organic compounds due to changing color when exposed to such vapors. Here, stability and the vapor sensing properties of butterfly and moth wings were investigated by optical spectroscopy in the presence of water vapor. It was shown that repeated 30 s vapor exposures over 50 min did not change the resulting optical response signal in a time-dependent manner, and after 5-min exposures the sensor preserved its initial properties. Time-dependent response signals were shown to be species-specific, and by using five test substances they were also shown to be substance-specific. The latter was also evaluated using principal component analysis, which showed that the time-dependent optical responses can be used for real-time analysis of the vapors. It was demonstrated that the capability to detect volatile organic compounds was preserved in the presence of water vapor: high-intensity color change signals with short response times were measured in 25% relative humidity, similar to the one-component case; therefore, our results can contribute to the development of biological photonic nanoarchitecture-based vapor detectors for real-world applications, like living and working environments.

## 1. Introduction

Natural photonic nanoarchitectures can be used to detect volatile organic compounds (VOCs), as the capillary condensation of vapors results in a reversible and reproducible color change [1,2]. These structures can be found in plants [3,4,5], aquatic animals [6,7,8], birds [9,10,11,12], and in the cuticles of butterflies and beetles [13,14,15]. The optical response of the biological sensor materials is fast and chemically selective, and it can operate at ambient atmospheric pressure and room temperature [16,17,18]. Therefore, these photonic nanoarchitectures occurring in living organisms have recently been used as multivariate VOC sensors directly [16,17,18,19,20,21,22,23,24] or as a basis of bio-inspiration [24,25,26,27,28,29,30,31].

The efficient detection of VOCs is crucial, as in our everyday indoor activities and living environments their presence is inevitable [32]. Additionally, VOCs have gained attention in medical diagnostics, being promising indicators for cancer and other diseases [33,34,35,36]. Therefore, it is important to develop sensors that can work in atmospheric humidity similar to our everyday living and working conditions, can endure repeated vapor exposures, and can be used for real-time analysis while remaining sensitive and chemically selective. The biological photonic nanoarchitecture-based sensors are excellent candidates for this task as they are cheap and ready-made devices produced at a macroscopic size in high quality, therefore they can be used in the potential applications instantly. Although their mass production does not seem trivial, they are still suitable as prototypes to the experiments where artificial materials are not currently available. The information obtained through these can be used to design bio-inspired photonic nanoarchitectures that are compatible with the requirements of mass production.

In our previous research, we have investigated the vapor sensing properties of several polyommatine butterfly species with nanoporous photonic nanoarchitectures in their wing scales [18]. Due to the open structure of the nanoarchitectures [37], the structural colors of these chitin–air nanocomposites changed rapidly when the vapor composition of the ambient atmosphere changed [38]. By measuring this optical response, the nature and concentration of the test volatiles could be determined [18]. It was found that during vapor exposure, capillary condensation took place, and due to the condensed liquid layer, swelling of the chitin nanoarchitecture occurred [17,18,39]. This resulted in both a substance-specific optical response and higher-than-expected sensitivity, which also could be tuned in a controlled way [40]. We have found that single wing scales can also detect VOCs with the same efficiency as whole wings [41]. Finally, the complex sensing mechanism, based on condensation and the swelling, allowed the detection of binary vapor mixtures [42], which provided the basis for detecting VOCs in atmospheric humidity.

In this study, the vapor-sensing properties of butterfly and moth wings were investigated by optical spectroscopy during numerous vapor exposure/purge cycles and in the presence of water vapor. We studied the effect of repeated short pulses and one single, long exposure of VOCs. It was found that the resulting optical response was reproducible and, in both cases, returned to its initial value after synthetic air purging. We also examined how the color change signal evolved during vapor exposure and found that the time-dependent optical response was substance-specific. When constant humidity conditions were applied, the wing-based sensors preserved their original VOC-sensing capabilities, showing the possibility of utilization in real-life applications in the future.

## 2. Materials and Methods

The butterfly and moth species used in the present study were not subjected to any restrictions. The specimens were obtained from the curated collection of the Hungarian Natural History Museum. Male specimens of *Chrysiridia rhipheus* (Uraniidae) [2,43], *Heliophorus bakeri* (Lycaenidae: Heliophorini) [44], *Hypochrysops polycletus* (Lycaenidae: Luciini) [45], *Morpho aega* (Nymphalidae: Morphini) [2,46], and *Polyommatus icarus* (Lycaenidae: Polyommatini) [37,47] species were investigated. The vapor-sensing capabilities of these species on binary vapor mixtures were recently studied [42]. These butterflies and day-flying moths reflect a whole spectrum of intense structural colors on their dorsal wing surfaces, which is generated by photonic nanoarchitectures located in the lumen of their wing scales. The open structure of these nanoarchitectures makes possible the fast exchange of test substances during vapor-sensing experiments, which results in a response time on the order of seconds.

**Figure 1:**
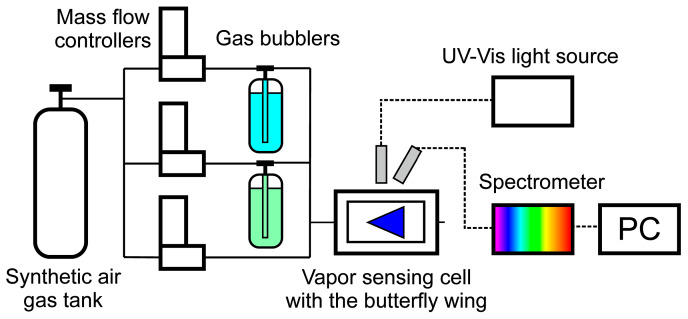
Schematic drawing of the measurement setup used in the vapor sensing experiment. Digital mass flow controllers were used to set the concentration of the vapor mixtures by letting synthetic air through the gas bubblers (blue: water, green: volatile component) in the desired ratio to the vapor sensing cell which contained the butterfly wing. The optical response of the wings was measured by a modular spectrophotometer.

As it was reported earlier [18,42], a custom air-proof aluminum cell with a quartz window, with a gas inlet and outlet on opposite sides, was used in the vapor-sensing experiment (Figure 1). The wings were inserted in the cell, and their optical response was measured when the test substances were let through the vents. The saturated vapors for the mixtures used were generated in gas bubblers. The desired vapor concentration was set by computer-controlled digital mass flow controllers (Aalborg DFC, Aalborg Instruments and Controls Inc., Orangeburg, NY, USA). A constant flow of 1000 mL/min of synthetic air (Messer, Bad Soden, Germany) carrier gas was used. The concentration of the test vapors was set to 50% (50% saturated vapor + 50% synthetic air) in the water vapor-free measurements. The water vapor concentration was set to 25% when constant humidity was applied, and 50% of the test vapors were used for the sensing measurements.

The samples were illuminated through the quartz window of the cell by an Avantes DH-S-BAL (Avantes BV, Apeldoorn, The Netherlands) UV-Vis light source (deuterium–halogen light source) under normal incidence. To avoid mirror-like backscattering from the quartz window, the reflected light was collected under ~45° with an Avantes HS 1024*122TEC spectrophotometer (Avantes BV, Apeldoorn, The Netherlands). The optical responses of the butterfly or moth wings were characterized by the color change during vapor exposure by defining the relative reflectance spectrum ∆R = (R/R_0_) × 100% [1]. The initial spectrum of the wings in artificial air (R_0_) was used as a reference during the vapor sensing measurements.

The test substances (acetone, chloroform, ethanol, and 2-propanol) used in the vapor-sensing experiments were reagent-grade and were obtained from VWR International Ltd. (Radnor, PA, USA).

Data processing and the evaluation of the measured reflectance data and the principal component analysis (PCA) were conducted using OriginLab OriginPro 2018 software (OriginLab Co., Northampton, MA, USA). PCA is a commonly used multivariate method that allows the finding of the highest possible variance of a data set [48]. Therefore, it is a very well-suited tool to separate the optical responses of the different test substances in the vapor-sensing measurements [1,18,21].

## 3. Results

**Figure 2:**
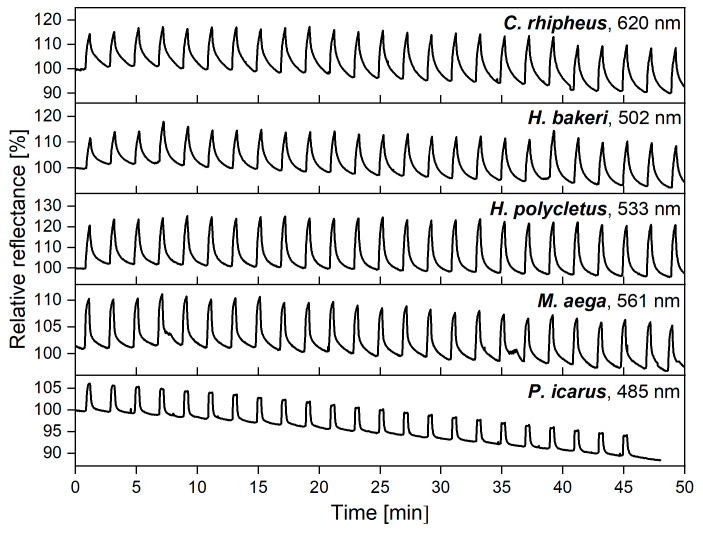
Vapor pulses (30 s, 50% ethanol) were applied to the wings of five investigated species, which was followed by 90 s purging with synthetic air. The process was repeated more than 20 times, and the maximum of the spectral response was plotted as a function of time. The nanometer values in the top right corners show the wavelength of the maximal spectral responses from which the time-dependent signals were generated.

Long-term, reliable operation is a key parameter when sensors are planned to be used in real-world applications. Therefore, reproducibility of the optical response was investigated by applying VOCs repeatedly on the wings of the five investigated species. First, 50% ethanol vapor (50% saturated ethanol vapor + 50% synthetic air) was applied for 30 s, which was followed by 90 s purging of the cell using synthetic air carrier gas, and this was repeated for 50 min. The measured relative reflectance spectra were recorded every second, and the maximal intensities of the spectral changes [38] were plotted as a function of time in Figure 2. During a typical vapor sensing measurement, the whole spectrum of the optical response is recorded as a function of time. This can be plotted as a three-dimensional surface which contains all the recorded data, similar to Figure 2 in [38]. To facilitate comparison, a single notable wavelength, e.g., where the maximum changes were measured, can be selected and plotted as a function of time, therefore two-dimensional graphs are made. Thusly, generated Figure 2 depicts more than 20 cycles for each sample during the 50-min measurement. The intensity of the spectral response was found to be independent from the number of exposures, and, with the exception of *P. icarus*, only slight variations and a minimal linear drift were observed during the measurements.

**Figure 3:**
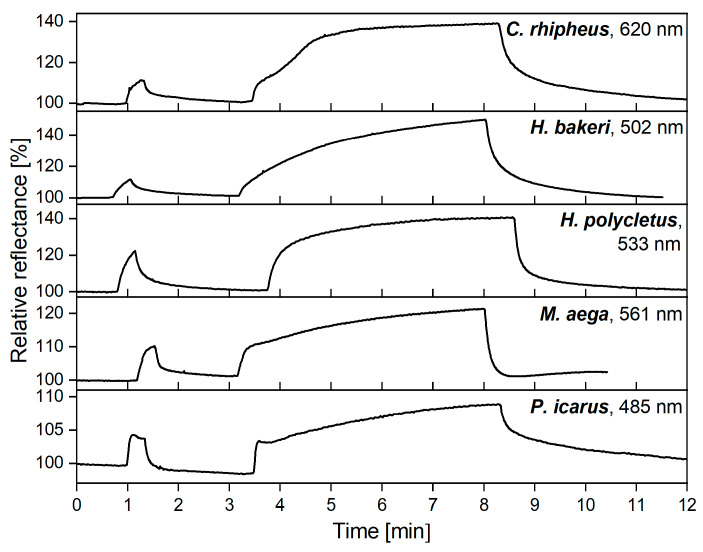
The first 30 s 50% ethanol vapor pulse was followed by a 5-min exposure, which resulted in saturation of the wing-based vapor sensors. After purging with synthetic air, the initial colors of the wings were retained. Both the short and long durations show specific time-dependent optical responses for the five samples. The nanometer values in the top right corners show the wavelength of the maximal spectral responses from which the time-dependent signals were generated.

The vapor sensors can be affected not only by periodic short pulses but also by a single, long exposure to VOCs. To investigate the effect of longer exposures, the first 30 s 50% ethanol vapor pulse was followed by a 5-min exposure (Figure 3). During this time, the response signal tended towards the saturation values in all cases. After the end of vapor exposure, during synthetic air purging, the response signals returned close to their initial values which were measured after the 30 s vapor exposure.

**Figure 4:**
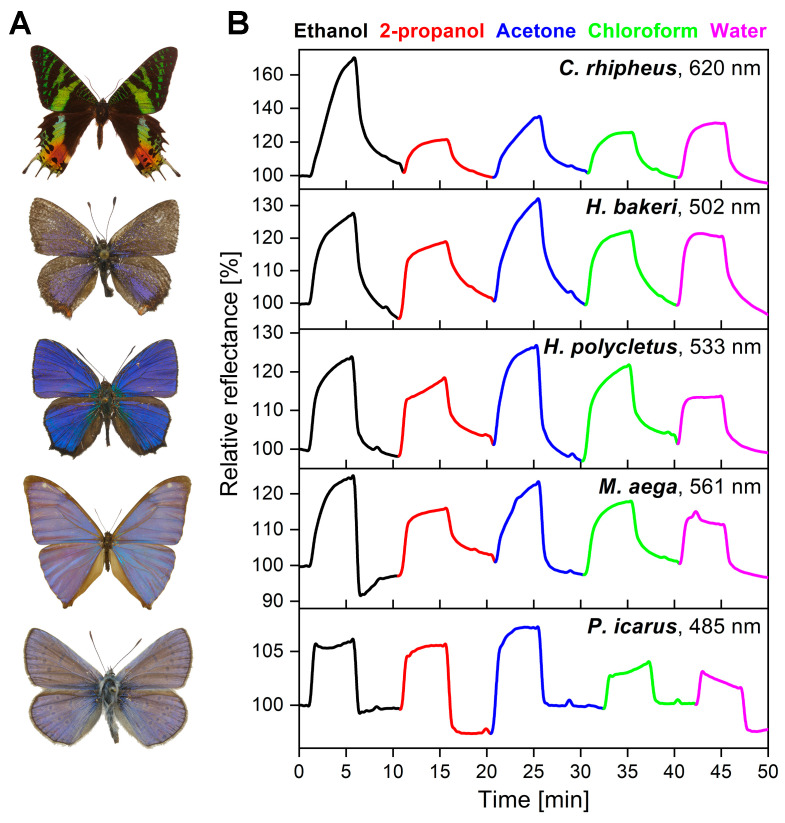
(**A**) Photographs of the investigated male specimens (not to scale). (**B**) Test substances were exposed to the wing-based sensors for 5 min, with 5-min purging in between. The measured time-dependent intensity of the spectral changes was plotted as a function of time. Different volatile organic compounds (VOCs) are noted by different colors. Each of the wings showed specific time-dependent optical responses for the five test substances. The nanometer values in the top right corners show the wavelengths of the maximal spectral responses from which the time-dependent signals were generated.

Figure 3 shows that both short and long pulses had characteristically different shapes for the five samples, suggesting that each photonic nanoarchitecture had species-specific, time-dependent optical response signals. To investigate whether the time-dependent signal is suitable for chemically selective sensing, four more test substances were used for 5-min vapor exposures on the wings in 50% dilution. The vapor flows were separated by 5-min purging of the cell with synthetic air. The maximum intensities of the spectral changes were plotted as a function of time in Figure 4.

**Figure 5:**
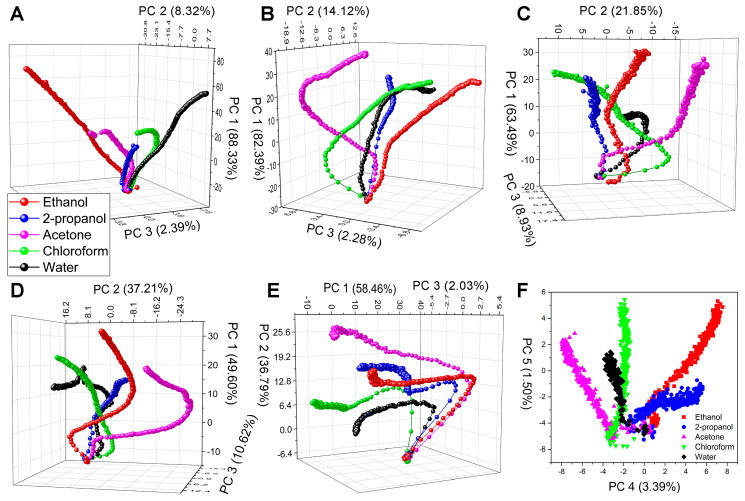
Principal component analysis (PCA) scores plots of the time-dependent vapor-sensing results of (**A**) *C. rhipheus*, (**B**) *H. bakeri*, (**C**) *H. polycletus*, (**D**) *M. aega*, and (**E**) *P. icarus*. The variation contributions of the first 3 PCs are indicated on the axes. (**F**) In the case of *H. polycletus*, where the cumulative variance of the first 3 PCs was below 95%, PC 4 and PC 5 were also tracking their paths as a function of time. The exposure time increases from the bottom to the top of the graphs when using 50% concentration of the test substances. All samples have individual, separated trajectories for the test VOCs showing the substance-selective sensing capabilities of the time-dependent measurement method and foreseeing its potential in real-time data processing.

The optical responses of the different test substances are indicated with different colors for all five samples. There were clearly visible differences in the shapes of the substance-specific signals between the VOCs, while the species-specific sensing properties were retained, suggesting that the time-dependent color change signal could also be used in substance-selective vapor sensing. Therefore, PCA evaluation of the time series with 1 s resolution was conducted for all test species and substances. Here, the rising sections of the time-dependent optical responses in Figure 4 were used as the input for the PCA. Figure 5 depicts the PCA scores plots of the five samples. It was found that the first three PCs cumulatively retained the variation of the original dataset more than 90%. All test substances had individual trajectories showing the substance-selective sensing capabilities of the measurement method based on the time-dependent vapor sensing signal. Furthermore, we found that in the case of *H. polycletus*, where the cumulative variance of the first 3 PCs was below 95%, PC 4 and PC 5 were also tracking their paths as a function of time.

**Figure 6:**
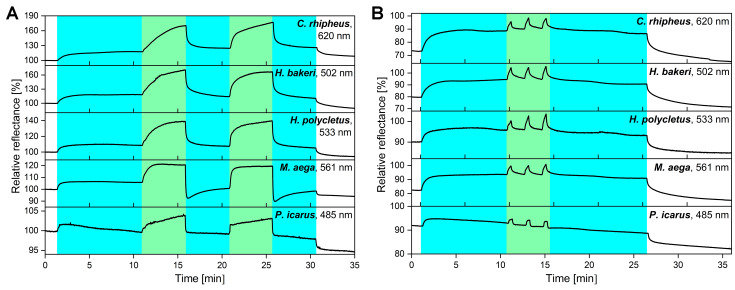
The VOC-sensing properties of the samples were tested in the presence of humidity. Therefore, a constant 25% water vapor background was applied, and it was followed by (**A**) two 5-min-long 50% ethanol vapor exposures or (**B**) three 30 s 50% ethanol pulses. In both cases, the response signal was separated from the background of the humidity, and the intensity of the time-dependent signal was preserved compared to the humidity-free case (Figure 3). The nanometer values in the top right corners show the wavelength of the maximal spectral responses from which the time-dependent signals were generated.

After the long-term vapor exposure measurements, the vapor-sensing properties in the presence of water vapor were investigated to simulate a real-world environment in the laboratory. Recently, we have shown that the photonic nanoarchitectures occurring in the wing scales of butterflies and moths are capable of chemically selective sensing when binary vapor mixtures are used [42]. Based on this, the VOC-sensing capabilities of the five test species were studied when a constant water vapor concentration was set in the sensing cell. Thus, a simulated environment was designed that reproduced the characteristics of the real-world environment: room temperature, atmospheric pressure, and in this case 25% relative humidity. The maximal intensity of the spectral change was plotted as a function of time in Figure 6, while 50% ethanol pulses were added to the constant water vapor background.

The wings placed in the vapor-sensing cell were first purged with synthetic air to clean their surfaces (white background in Figure 6). After a few minutes, the water vapor flow at 25% concentration was initiated and continued for 30 min (blue background). The first 50% ethanol exposure was started after 10 min, the second one after 20 min (green background), and both were 5 min long (Figure 6A). They were followed by 5-min only water vapor exposures. As a final step, the sensing cell was purged with synthetic air, and the initial color of the wings was retained, with the exception of *P. icarus* for which a small downshift of the curve may be observed.

In the second measurement, short ethanol pulses were applied to investigate whether the sensors preserved their short response times and relatively high sensitivities for VOCs in the presence of humidity. Therefore, the duration of the ethanol pulses was set to 30 s and repeated three times (Figure 6B). In both cases, the response signal for ethanol was well-separated from the background of the water vapor. Furthermore, the intensity was not affected by humidity; almost as high signals were measured as in the humidity-free case (Figure 3).

## 4. Discussion

In order for VOC detectors based on photonic nanoarchitectures of biological origin to have medical or environmental monitoring applications, the following properties are essential: short response time, applicability at room temperature and atmospheric pressure, chemical selectivity, and reproducible and reliable operation in the presence of humidity. The first three attributes are inherent in natural photonic nanoarchitectures, as their open chitin–air structure allows rapid exchange of ambient vapors in standard conditions. Chemical selectivity was demonstrated using both one-component and binary mixtures in the wings of several butterflies and moths [1,2,18,20,42]. In this study, the reproducibility, stability, and operation in the presence of humidity were examined.

In the case of reproducible operation, it is important to investigate two different aspects of vapor exposure and determine how they affect detection. One is the case of repeated short pulses, the other is the saturation of the sensor materials. When 30 s ethanol vapor pulses were applied, all five test specimens showed similar behaviors (Figure 2). The rapid rise of the signal started immediately after the vapors were introduced and was preserved until synthetic air purging. For over more than 20 cycles (50 min) the maximum height of the optical response did not change; only slight linear drift may be observed. The same characteristic peak can be observed in Figure 3 where only one single 30 s pulse was applied in the first step. The similarity of the peaks in the two measurements showed that repeated exposure did not affect operation of the wing-based sensor.

In our recent work, we have used these short pulses for substance-selective vapor sensing, as the wing-based detectors have characteristic optical responses during this short response time [18]. However, the spectral changes were also found to be time-dependent, which was not utilized in that study. Therefore, we investigated the effect of longer vapor exposures, which revealed the spectral reflectance change of the wings near the saturation regime when a single 5-min vapor exposure was applied (Figure 3). Characteristic differences in the time-dependent optical responses also can be observed, which were consistent with our previous results [18] where the relative reflectance spectra were shown to be species-specific. Here, we investigated the progression of the maximum value of the optical response, and we found that the species-specific characteristics were preserved. This is an important feature because real-time processing of vapor-sensing data requires the time-dependent response signals of each material to be characteristically different from each other. It is also important to avoid too high vapor concentrations, as at a certain temperature rapid condensation of the vapors may occur, which changes the structural color drastically [49] and also makes vapor detection impossible.

Substance-specific detection can be realized in real time if response signals are characteristically different not only between species, but also between substances. Therefore, we also examined how time-dependent signals developed for five VOCs during long exposures (Figure 4). We found that the shape and rise of the signals varied from substance to substance, which also could be verified by PCA to show that individual vapors produced unique time-dependent signals as every test substance had distinct trajectories in the PCA space when the first 3 PCs were used in the scores plots (Figure 5A–E). Furthermore, when the cumulative variance of the first three PCs was below 95% in the case of *H. polycletus*, the PC4–PC5 scores plot also showed the substance selective behavior as distinct trajectories were found (Figure 5F) showing the five-dimensional dispersion of the optical response. These are consistent with our previous results [18,42], but provide an important addition: a well-defined wavelength of the entire measured spectrum also inherits its unique properties, and the possibility of vapor-selective sensing, if its time-dependence is also taken into account; thus, this time-dependence could be used as a “fingerprint” of that substance. This can be explained if we consider that each VOC interacts differently with the chitin nanoarchitectures. The rates of capillary condensation will be different, and the chitin nanoarchitecture will swell differently due to the condensed fluids, which results in substance-specific time-dependent optical responses. This inherent multivariate behavior of wing-based vapor detection is currently unique compared to other artificial vapor sensors, as such chemical selectivity can only be achieved by appropriate functionalization methods.

Finally, we examined the VOC-sensing capabilities of the wing-based sensors in the presence of water vapor (Figure 6). We found that similar changes occurred during the process in all specimens, independent of the structure of their photonic nanoarchitectures: water vapor produced a continuous background, while the signal of ethanol was not suppressed by this but was added to it in both 30 s pulses and 5-min exposures. This shows that by choosing a suitable reference level, the effect of continuously present water vapor can be taken into account. Moreover, air humidity measurements have well-established methodology and instrumentation. If the humidity level in artificial air is selected as a reference, the quantity of other substance can be measured directly, while if we measure with a synthetic air reference, both humidity and the concentration of the test substance can be deduced. Presumably, this is caused by the combination of capillary condensation and conformal deformation of the photonic nanoarchitecture: swelling of the chitin structure, caused by water vapor and ethanol together after their condensation, develops in such a way that swelling by water is enhanced in the presence of ethanol and, thus, increases the measured optical response. This provides an opportunity not only to analyze pre-measured data sets, but also to perform real-time measurements in the presence of water vapor based on the substance-specific time-dependence of the color change signal.

## 5. Conclusions

Photonic nanoarchitectures occurring in the wing scales of butterflies and moths were found to be efficient multivariate sensors for one-component and binary mixtures of VOCs as their optical response was fast and chemically selective, while they could operate at room temperature and atmospheric pressure. In this study, the reproducibility, stability, and operation in the presence of water vapor were investigated. It was found that series of short vapor pulses resulted in similar optical responses, and after a long exposure the wings preserved their initial sensing properties. We demonstrated that the wing-based sensors had species-specific time-dependent optical response signals, and by using five test VOCs they were shown to be substance-specific. Principal component analysis of the time-dependent data showed that the optical responses can be used for real-time analysis of the vapors. We demonstrated that the sensors were capable to detect VOCs in the presence of humidity similar to the one-component case, as high-intensity optical response signals with short response times were measured in the presence of water vapor. Based on these, VOC sensors can be made from the wings of butterflies and moths that can endure long-term operation in atmospheric humidity and are capable of real-time analysis that meet the requirements of the potential applications, such as medical diagnostics or monitoring indoor activities and living environments.
